# Antiepileptic Drug Nonadherence and Its Predictors among People with Epilepsy

**DOI:** 10.1155/2016/3189108

**Published:** 2016-12-08

**Authors:** Asmamaw Getnet, Solomon Meseret Woldeyohannes, Lulu Bekana, Tesfa Mekonen, Wubalem Fekadu, Melak Menberu, Solomon Yimer, Adisu Assaye, Amsalu Belete, Habte Belete

**Affiliations:** ^1^Finote Selam Hospital, Finote Selam, Ethiopia; ^2^Diseases Prevention and Control, School of Public Health, University of Queensland, Brisbane, QLD, Australia; ^3^Amanuel Mental Specialized Hospital, Addis Ababa, Ethiopia; ^4^College of Medicine and Health Sciences, Bahir Dar University, Bahir Dar, Ethiopia; ^5^College of Health Sciences, Mizan Tepi University, Mizan, Ethiopia; ^6^Psychiatry Department, College of Health Sciences and Medicine, Dilla University, Dilla, Ethiopia; ^7^Debre Markos University, Debre Markos, Ethiopia; ^8^College of Medicine and Health Sciences, Debre Tabor University, Debre Tabor, Ethiopia

## Abstract

*Introduction.* Antiepileptic drugs are effective in the treatment of epilepsy to the extent that about 70% of people with epilepsy can be seizure-free, but poor adherence to medication is major problem to sustained remission and functional restoration. The aim of this study was to assess the prevalence and associated factors of antiepileptic drug nonadherence.* Methods.* Cross-sectional study was conducted on 450 individuals who were selected by systematic random sampling method. Antiepileptic drug nonadherence was measured by Morisky Medication Adherence Scale (MMAS) and logistic regression was used to look for significant associations.* Result.* The prevalence of AEDs nonadherence was 37.8%. Being on treatment for 6 years and above [AOR = 3.47, 95% CI: 1.88, 6.40], payment for AEDs [AOR = 2.76, 95% CI: 1.73, 4.42], lack of health information [AOR = 2.20, 95% CI: 1.41,3.43], poor social support [AOR = 1.88, 95%, CI: 1.01, 3.50], perceived stigma [AOR = 2.27, 95% CI: 1.45, 3.56], and experience side effect [AOR = 1.70, 95% CI: 1.06, 2.72] were significantly associated with antiepileptic drug nonadherence.* Conclusion.* More than one-third of people with epilepsy were not compliant with their AEDs. Giving health information about epilepsy and its management and consequent reduction in stigma will help for medication adherence.

## 1. Introduction

Epilepsy is a chronic disorder of the brain and is one of the most common serious neurological disorders affecting 50 million people worldwide with no boundary to age, race, social class, nationality, or geographical location [1, 2]. Among patients who had epilepsy, 85% of them found in developing countries and estimated 40 million people do not receive appropriate treatment [1, 3]. The overall mortality rate due to epilepsy increased by twofold to threefold as compared with the general population [4]. Medication nonadherence is a voluntary or involuntary behavior of medication intake which includes failing initially filling or refilling a prescription, discontinuing a medication before the course of therapy is completed, inability to adhere with agreed recommendations from health care provider, taking more or less of a medication than prescribed, and taking a dose at wrong time [5, 6].

The magnitude of antiepileptic drug nonadherence is ranged from 26% in USA to 67% in Nigeria [7, 8]. As a study done in the North Carolina indicated, the prevalence of AEDs nonadherence was 39% and it was higher (43%) in elderly accompanied with increased likelihood hospitalization [9]. A primary care based study in UK showed that the prevalence of antiepileptic drug nonadherence was 36.4% and those who were on multidrug treatment were tend to be noncompliant with their treatment [10]. A study conducted in Finland indicated the prevalence of AEDs nonadherence as 34% and nonadherence was higher in individuals who smoke cigarette and drink alcohol [11]. In sub-Saharan African countries, prevalence of antiepileptic drug nonadherence is significant which was about 67% in Nigeria, 54% in Kenya, and 37% in Ethiopia and financial factors were the significant predictors of nonadherence [8, 12–14].

Antiepileptic drugs (AEDs) are effective in the treatment of epilepsy, but poor adherence to medication is major problem to sustained remission and functional restoration resulted in treatment failure and seizure recurrence [15, 16]. Even though around 70% of people who had epilepsy supposed to be seizure-free with optimum AED treatment, many people with epilepsy did not take their antiepileptic drugs appropriately and the mortality rate in nonadherent patients was more than threefold higher than that of adherent one [17–19]. The consequence of AEDs nonadherence behavior has been associated with poor seizure control, increased morbidity and mortality along with increased time of hospitalization, worsened patient outcome, poor quality of life, and increased health care cost [20–23]. AEDs nonadherence will also lead to increase burden of inpatient and emergency department services; moreover, it also affects the family members socially, economically, and psychologically [3, 7, 14, 24].

There is scarcity of published information regarding the prevalence and associated factors of AEDs nonadherence in Ethiopia. A study which attempted to fill this information gap and came up with strong recommendation on possible interventions for improving medication adherence behavior among people with epilepsy is very important. Poor adherence to antiepileptic drugs is one of many reasons for pharmacological treatment failure and recurrence of seizure and consequently results in poor quality of life, decreased productivity, and seizure related social and economic crisis. Therefore this study was aimed at assessing the prevalence and associated factors of antiepileptic drug nonadherence among people with epilepsy.

## 2. Methods

Institution based cross-sectional study design was conducted from May 11 to June 8, 2015. The study was conducted on two hospitals in Northwest Ethiopia (Debre Markos Referral Hospital and Finote Selam District Hospital). Both hospitals provide service for more than 3.6 million population and there were around 1120 people with epilepsy who had follow-up in both hospitals. Adult individuals (18 and above years of age) who were diagnosed for epilepsy and have been on AEDs treatment for at least three months were included in the study and individuals who were unable to communicate due to serious neurological deficit and other serious illness were excluded.

The total sample for the study was 459, proportionally allocated for both hospitals (274 for Debre Markos Referral Hospital and 185 for Finote Selam District Hospital) and selected by systematic random sampling technique. The outcome variable for this study was antiepileptic drug nonadherence and the independent variables were as follows: sociodemographic variables: age, sex, marital status, ethnicity, religion, wealth index, educational status, occupation, and place of residence; patient and treatment related factors: perceived stigma, substance use, social support, AED type, drug side effects, duration of treatment, comorbid illness, and number of medications.

Data were collected by interviewing participants and reviewing patient charts using structured and pretested questionnaire by trained data collectors. AEDs nonadherence was measured by an eight-item Morisky Medication Adherence Scale (MMAS). A modified MMAS-8 item was developed from the original MMAS-4 during 2008 by Morisky and his colleagues. The first seven items are dichotomous response categories with yes or no and the last item is five-point Likert scale response [12, 25–27]. Social support was assessed by using the Oslo 3-items social support scale; the first item is 4-point Likert scale and the other two items are 5-point Likert scale. The sum score of this scale is ranged from minimum of 3 to the maximum 14, which is categorized into poor support 3–8, moderate support 9–11, and strong support 12–14 [28]. Felt stigma towards epilepsy was measured by using Kilifi stigma scale of epilepsy and the score above 10 indicated presence of perceived stigma [29].

The coded data entered into EPI INFO software version 3.5.3 and exported to SPSS version 20 for analysis. Descriptive statistics were used to describe the data. Bivariate and multivariable logistic regression were used to ascertain the association between covariates and the dependent variable. Odds ratios along with the 95% confidence intervals were used to show the strength of associations and *p* value of less than 0.05 was considered as statistically significant. Regarding wealth index analysis, variables which measure the urban and rural communities were selected and the frequency was checked and those variables with less than 10 frequencies were avoided. Principal component analysis was used and variables which extraction values < 0.5 were excluded step by step starting from small extraction value until extraction value ≥ 0.5. Finally all variables with extraction value of ≥ 0.5 were analyzed in sum factors and rank wealth index according to Ethiopia Demographic and Health Survey [30].

Ethical clearance was obtained from University of Gondar and Amanuel mental specialized hospital Institutional Review Board (IRB). Participants were assured that if they want to refuse to participate, their care or dignity would not be compromised in any way since there was no relationship between participation and health or treatment outcome. Confidentiality was maintained by anonymous questionnaire and informed consent was obtained from each participant.

## 3. Result

From the total of 459 sample size, 450 participants were interviewed with the response rate of 98.04%. The other 9 participants refuse the interview and are considered as nonrespondent. Among the study participants, 264 (58.7%) were males and the median age of participants was 27 years with interquartile range 14. All of the participants were Amhara in ethnicity and 393 (87.3%) were orthodox in religion. From total participants, 206 (45.8%) were single followed by 167 (37.1%) married. More than half of the participants 274 (60.9%) were self/private employed and 201 (44.7%) participants were unable to read and write. From the participants, 87 (19.3%) and 68 (15.1%) had lowest and highest wealth index respectively, and majority of respondents 338 (75.1%) predominantly resided in the rural areas (see Table 1).

## 4. Clinical and Patient Related Factors

Majority of the respondents, 345 (76.7%), were on monotherapy and Phenobarbital was the most commonly prescribed AED. Comorbid illness (depression, HIV, dyspepsia, schizophrenia, and asthma) was reported by 28 (6.2%) participants and majority of the participants 329 (73.1%) were treated for epilepsy for at least 2 years. More than one-third of the participants reported that they did not get health information about their condition and side effects of AEDs; and 127 (28.2%) of the participants experienced side effect of AEDs, most frequently sedation. Perceived epilepsy related stigma was also experienced by 34.9% of people with epilepsy (see Table 2).

## 5. Prevalence and Associated Factors of Antiepileptic Drug Nonadherence

The overall prevalence of antiepileptic drug nonadherence among the study participants was 37.8%. During multivariate analysis of AEDs nonadherence in relation to all associated variables, ways of getting medications (free of charge or with payment), status of getting health information from health care providers, social support, perceived epilepsy related stigma, presence of antiepileptic medication side effects, and duration of the treatment were significantly associated (see Table 3).

## 6. Discussion

Nonadherence to treatment is one of many reasons for pharmacological treatment failure and seizure recurrence. AEDs nonadherence was assessed by an eight-item Morisky Medication Adherence Scale. Its sensitivity and specificity were 93% and 53%, respectively, and the participant was considered as nonadherent to AEDs if the score of MMAS was 2 or more scales [25, 26]. In this study, the prevalence of antiepileptic drug nonadherence among people with epilepsy was 37.8% with 95% CI (33.3%; 42.3%).

The finding of this study was consistent with the studies conducted in North Carolina 39%, Finland 34%, and UK 36.4% [9–11]. However, it was greater than the study done in USA 26% and the probable explanation might be the differences in study design (retrospective cohort in USA) and socioeconomic differences [7]. The finding of this study was lower than the studies done in Brazil, Nigeria, and Palestine which were 66.2%, 67.4%, and 64%, respectively, and this difference was probably due to the difference in AEDs multidrug treatment. For instance, 71.1% of people with epilepsy in Brazil, 85% in Nigeria, and 63.2% in Palestine were on multiple AEDs treatment while in the current study, only 23.3% of people with epilepsy were in poly-AEDs treatment [25, 31, 32].

People with epilepsy who were buying AEDs were about 2.76 times more likely as to be nonadherent as compared with those who were getting their AEDs free of charge [AOR = 2.76, 95%, CI: 1.73, 4.42]. The long-term nature of epilepsy treatment might contribute for this association; buying medications for long period of time lets the individual to feel bored about his condition and consequently end up in nonadherence. The finding was supported by the study from Kenya [12]. Individuals who did not get health information about their illness, duration of treatment, and drug side effect were about 2.2 times more likely to be nonadherent than their counterparts [AOR = 2.20, 95%, CI: 1.41, 3.43]. Unless they got sufficient information, people with epilepsy tends to stop taking AEDs immediately after the seizure has been controlled or whenever they experience side effects [13].

Concerning social support, participants who had poor social support were 1.88 times more likely to be nonadherent as compared with those who had strong social support [AOR = 1.88, 95%, CI: 1.01, 3.50]. Favorable perception for social support had positive correlation to be adherent with drugs and this finding was supported by other studies [13, 33]. Regarding duration of treatment, those people with epilepsy who were being on treatment for 6 years and above were 3.47 times more likely to be nonadherent as compared with participants who were on treatment for 3 months to 1 year [AOR = 3.47, 95%, CI: 1.88, 6.40] and being on treatment for 2–5 years who were 2.32 times more likely to be nonadherent as compared with their counterparts [AOR = 2.32, 95%, CI: 1.31, 4.10]. The present study showed that as treatment duration increases, the participants became more likely to be nonadherent and this is supported by other studies [12, 13].

The odds of being nonadherent for participants who had perceived epilepsy related stigma were 2.27 times [AOR = 2.27, 95%, CI: 1.45, 3.56] as compared to those who had not. The actual physical act of having to take medication can increase the levels of stigma experienced by the person with epilepsy and taking AEDs reminds the individuals that they have epilepsy and they may keep pill-taking in public to the minimum resort [34, 35]. Those participants who had AEDs side effect were 1.70 [AOR = 1.70, 95%, CI: 1.06, 2.72] times more likely to be nonadherent as compared with those who had no AEDs side effect. As indicated by different studies, most complaints of people with epilepsy are related with medication side effects and is probably the commonest cause for discontinuing AEDs without consulting the health care giver [35, 36].

## 7. Conclusion

The prevalence of antiepileptic drug nonadherence among patients with epilepsy was high. AEDS nonadherence was significantly associated with variables like availability of health information, epilepsy related stigma, presence of social support, AEDs side effects, duration of the treatment, and ways of getting AEDs (free of charge or with fee). Giving brief psychoeducation about epilepsy, AEDs side effect and the importance of sticking with the recommended drug use can improve AEDs adherence. Information dissemination to the people with epilepsy and to the public at large is important to prevent AEDs nonadherence and to promote healthy life for those individuals.

## Figures and Tables

**Table 1:** Sociodemographic characteristics of people with epilepsy, Ethiopia, 2015 (*n* = 450).

Variables	Categories	Frequency (*n*)	Percent (%)
Sex	Male	264	58.7
	Female	186	41.3

Age	18–25	210	46.7
	26–44	195	43.3
	45 and above	45	10

Marital status	Single	206	45.8
	Married	167	37.1
	Divorced	63	14
	Widowed	14	3.1

Religion	Protestant	27	6
	Muslim	30	6.7
	Orthodox	393	87.3

Ethnicity	Amhara	450	100

Occupation	Self/private employed	274	60.9
	Jobless	144	32
	Government employee	32	7.1

Educational status	Unable to read and write	201	44.7
	Primary school	149	33.1
	Secondary school	50	11.1
	Diploma and above	50	11.1

Wealth index	Lowest	87	19.3
	Second	91	20.2
	Middle	101	22.4
	Fourth	103	22.9
	Highest	68	15.1

Residence	Rural	338	75.1
	Urban	112	24.9

**Table 2:** Distribution of patients with epilepsy disorder by clinical and treatment related factors, Ethiopia, 2015 (*n* = 450).

Variables	Category	Frequency	Percent (%)
Current AEDs	Phenobarbital	408	90.7
	Phenytoin	64	14.2
	Sodium-valproate	30	6.7
	Carbamazepine	54	12

Number of AEDs prescribed	One	345	76.7
	Two	105	23.3

Comorbid illness	No	422	93.8
	Yes	28	6.2

Reported side effects	No	323	71.8
	Yes	127	28.2

Duration on treatment	3 months–1 year	121	26.9
	2 years–5 years	193	42.9
	6 years & above	136	30.2

Getting medication	Freely	165	36.7
	By payment	285	63.3

Getting health information	Yes	289	64.2
	No	161	35.8

Ever substance use	No	405	90
	Yes	45	10

Current substance use	No	428	95.1
	Yes	22	4.9

Perceived stigma	No	293	65.1
	Yes	157	34.9

Social support	Poor social support	128	28.4
	Moderate social support	219	48.7
	Strong social support	103	22.9

**Table 3:** Factors associated with antiepileptic drug nonadherence among people with epilepsy, Ethiopia, 2015 (*n* = 450).

Independent variables	AEDs nonadherence		COR (95% CI)	AOR (95% CI)
	No	Yes		
Co morbid illness				
No	266	156	1	1
Yes	14	14	1.71 (0.79–3.67)	1.29 (0.53, 3.10)
Reported side effect				
No	216	107	1	1
Yes	64	63	1.99 (1.31–3.02)	**1.70 (1.06, 2.72)**
Getting medication				
Free	125	40	1	1
Payment	155	130	2.62 (1.71–4.01)	**2.76 (1.73, 4.42)**
Getting health information				
No	77	84	2.58 (1.73–3.84)	**2.20 (1.41, 3.43)**
Yes	203	86	1	1
Ever substance use				
no	258	147	1	1
Yes	22	23	1.84 (0.99–3.41)	1.35 (0.67, 2.73)
Duration on treatment				
3 months–1 year	96	25	1	1
2 years–5 years	117	76	2.49 (1.47–4.22)	**2.32 (1.31, 4.10)**
6 years and above	67	69	3.96 (2.27–6.88)	**3.47 (1.88, 6.40)**
Social support				
Poor social support	58	70	3.23 (1.85–5.64)	**1.88 (1.01, 3.50)**
Moderate social support	147	72	1.31 (0.78–2.20)	1.04 (0.59, 1.83)
Strong social support	75	28	1	1
Perceived stigma				
No	209	84	1	1
Yes	71	86	3.01 (2.01–4.51)	**2.27 (1.45, 3.56)**
